# Survival Benefit of Adjuvant Chemotherapy in Pulmonary Carcinoid Tumors

**DOI:** 10.3390/cancers14194730

**Published:** 2022-09-28

**Authors:** Philip T. Sobash, Asad Ullah, Nagla Abdel Karim

**Affiliations:** 1 Georgia Cancer Center, Augusta University, Augusta, GA 30912, USA; 2 Department of Pathology, Vanderbilt University Medical Center, Nashville, TN 37232, USA; 3 Inova Schar Cancer Institute, University of Virginia, Fairfax, VA 22031, USA

**Keywords:** pulmonary carcinoid, adjuvant chemotherapy, survival benefit, atypical carcinoid, neuroendocrine tumor

## Abstract

**Simple Summary:**

Chemotherapy administration after surgical resection of pulmonary carcinoid is not well studied. While guidelines state adjuvant therapy may be appropriate in some scenarios, this is not observed clinically. We reviewed the literature to examine if adjuvant chemotherapy in patients with resected pulmonary carcinoid provided any survival benefit. The aim of this review is to aid clinicians when deciding on next steps for these patients after resection.

**Abstract:**

Pulmonary carcinoid tumors are a rare subtype of neuroendocrine cell tumor found in approximately 1–2% of lung cancers. Management is primarily through surgical resection, with limited benefit of adjuvant therapy in the clinical setting. Genomic profiling is in the nascent stages to molecularly classify these tumors, but there are promising insights for future targeted therapy. A total of 80 abstracts were analyzed for further review with 11 included in our final analysis. Only 4 of the 11 reviewed in depth provided statistical analysis. We evaluated PFS, OS, 1- and 5-year survival as mentioned in the studies. Nodal and KI67 status were also analyzed. Based on the current literature, there is no definitive evidence that adjuvant chemotherapy after resection confers a survival benefit in typical or atypical carcinoids.

## 1. Introduction

Pulmonary carcinoid tumors are a rare type of neuroendocrine tumor (NET) comprising less than 1–2% of all lung cancers [1]. These tumors can be further subclassified into typical carcinoid (TC) (low grade) and atypical carcinoid (AC) (intermediate grade) [2]. Primary treatment consists of surgical resection [3]. Differing surgical techniques are utilized depending on the location and extent of the tumor. Incomplete resection is a poor prognostic indicator and can often lead to the locoregional recurrence of the disease [3,4,5].

The use of cytotoxic agents as adjuvant therapy is mixed and highly debated among clinicians. While guidelines state the use of adjuvant therapy may be appropriate in certain cases, there are a lack of high-quality data due to the rarity of the disease [6]. While there is some evidence for treatment of aggressive metastatic pulmonary carcinoid, the evidence for use after surgical resection is lacking [7]. There are currently no prospective or randomized control trials concerning treatment modalities in regard to pulmonary carcinoid status post-resection. Expert opinion and organizational recommendations concerning individuals with surgical resection are mixed and complicated by a lack of applicable data.

The European Neuroendocrine Tumor Society recommends the use of adjuvant chemotherapy for atypical carcinoids after surgical resection, with Level IV evidence [5]. This is consistent with the National Comprehensive Cancer Network (NCCN) guidelines for adjuvant therapy with a 2B classification of evidence [8]. The North American Neuroendocrine Tumor Society and European Society for Medical Oncology currently have no current recommendations concerning therapy [9,10]. This paper reviews and summarizes the existing literature on survival benefits of individuals who received adjuvant chemotherapy after surgical resection of pulmonary carcinoid.

## 2. Materials and Methods

MEDLINE/PubMed was utilized in accordance with PRISMA guidelines that identified the studies used to evaluate survival and use of adjuvant chemotherapy in typical and atypical carcinoids. Search parameters were limited from 2010 to 2021 and were evaluated further to meet inclusion and exclusion criteria.

### 2.1. Inclusion Criteria

- Study contains subjects who were surgically treated for well-differentiated typical or atypical carcinoid.
- Study must contain patients that received chemotherapy.
- Study must give a recommendation, whether qualitative or quantitative, concerning the benefit of chemotherapy if any.

### 2.2. Study Selection

A total of 80 studies were identified through the search parameters “chemotherapy” AND “pulmonary” AND “carcinoid”. The abstracts of these results were reviewed for possible inclusion in the study. Of the original articles reviewed, only eleven had reference to the survival benefit of our interest. These articles were analyzed in their entirety and of those. Four had statistical data reported to aid in their conclusions. Specific types of chemotherapy regimens used in trials were not necessarily clear and thus not assessed in this review. Of interest was a subgroup analysis of the nodal status and staging in concurrence with adjuvant therapy that was pre-specified before selection, but not an inclusion criterion.

### 2.3. Data Collection

Due to the heterogeneity of studies, outcomes of interest if applicable were: overall survival (OS), progression-free survival (PFS), median survival, and survival at 1 and 5 years. Exclusion criteria included abstracts, case reports, manuscripts not in English, and articles that did not specify if the primary surgical intervention had been employed before chemotherapy.

### 2.4. Study Risk Bias Assessment

Articles were reviewed by both authors independently and screened for the inclusion criteria aforementioned. Articles that did not meet criteria based on abstract review were excluded from the full analysis. The remaining articles were analyzed and grouped based on qualitative or quantitative. Due to the nature of the studies selected, the authors found it pertinent to include those with and without statistical analysis.

### 2.5. Additional Analyses

Additional criteria analyzed but not part of the selection criteria were nodal status and tumor stage on survival relative to chemotherapy use.

## 3. Results

### 3.1. Main Results

Using the terms “chemotherapy” AND “pulmonary” AND “carcinoid.”, there were 80 studies selected for further analysis for review. The initial review was an abstract review for further evaluation of article consideration. Articles that were not reported in english, and case reports were not included in this systematic review. Out of the initial 80 articles selected for pre-review, 11 were identified to meet the inclusion criteria. Of the 11 cases reviewed, 4 contained some sort of statistical analysis. Many initially reviewed studies did report conclusions and opinions regarding the main objectives, but they did not report findings by outcomes evaluated in this manuscript. Type of chemotherapy was reported in some but not all, of the studies; therefore, individual regimens were not discussed. The sample size in most studies reviewed did not have enough power for statistical analysis. Due to heterogeniety among the studies reviewed, outcomes were reported by: median survival, 1 and 5-year survival, PFS and OS. No study reported a survival benefit with adjuvant chemotherapy, with one reporting worse OS, versus observation in nodal vs. non-nodal disease or based on Ki-67 index.

### 3.2. Subgroup Analysis

**Figure 1:**
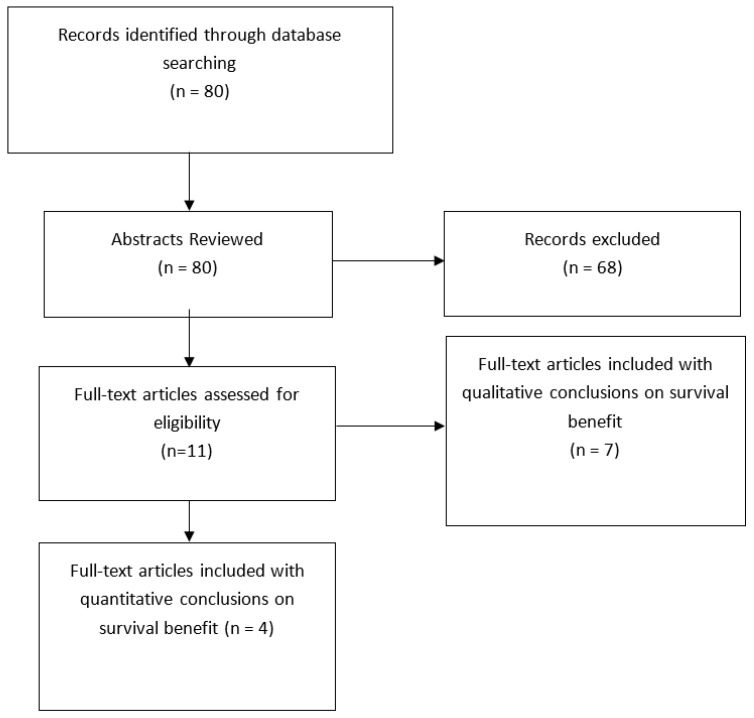
Study Selection—PRISMA Diagram. The systematic review followed the recommendations of the Preferred Reporting Items for Systematic Reviews and Meta-Analyses (PRISMA). The protocol has not been reg-istered.

Nodal status and staging appear to confer a disadvantage when adjuvant chemotherapy is employed. One study did demonstrate a percentage overall survival increase at 5 years in Stage III AC with chemotherapy use, but this was not statistically significant (Figure 1).

## 4. Discussion

### 4.1. Clinical Implications

#### 4.1.1. Survival Benefit with Adjuvant therapy

Ten of the eleven studies analyzed in this review did not conclude or recommend the use of adjuvant therapy in typical or atypical carcinoids that offered any survival benefit (Table 1). Tumor response was noted in multiple of the articles reviewed, which is supported by the literature. Our review did not directly analyze the different regimens used in the retrospective studies, and in many cases, these were not reported. In the article by Nussbaum et al., there was actually a statistically significant survival disadvantage conferred with adjuvant chemotherapy with typical carcinoids, although when propensity-matched, the statistical significance ceased.

Overall, there was fewer atypical than typical carcinoids seen throughout the studies. This lines up with current literature on the incidence. Three studies specifically analyzed typical and atypical, respectively [11,12,13]. In all three, there were no survival benefits with therapy, regardless of nodal status. Many studies examined a mixed cohort, and due to their retrospective nature were unable to properly delineate, or did not report, the breakdown between subtypes. Gosain et al. did review TC and AC in the same study and had one of the higher numbers of total individuals analyzed.

#### 4.1.2. Survival Benefit Based on Nodal Status, Ki-67 Index, and Metastasis

Two studies, Nussbaum et al. and Song et al., reported nodal status. In each study, N0 and N+ carcinoids did not differ in a survival advantage [13,14]. For staging, if any difference does exist, could be possibly exist for Stage III AC. This was evidenced by Gosain et al., who showed an OS benefit at 5 years with chemotherapy in Stage III disease [15]. These outcomes are consistent with the current guidelines from NCCN and European Neuroendocrine Tumor Society (ENETS) (Table 2), although it is of note the results were not statistically significant. There was a higher total number of patients analyzed with approximately 800 AC patients compared to many other reviews presented here. This is the most conclusive evidence in our review based on the power of the study.

Cytotoxic chemotherapy has been shown to give more of a response in pulmonary neuroendocrine tumors (pNETs) than non-pulmonary neuroendocrine tumors (non-pNETs) [22]. Multiple studies demonstrate that having a higher mitotic and Ki-67 index are poor prognostic indicators [23,24,25]. Contrarily, other studies examining Ki-67 index in conjuction with histopathology found no additional value for prognosis, but some value in prediction of recurrence [26,27,28]. Ki-67 may have more of a benefit in TC than AC. [26] Studies assessing Ki-67 as a response to chemotherapy have shown the higher the index the more responsive, in general, the tumor is [29]. These results from the various studies can be seen in Table 3. Overall, survival is not statistically significant for chemotherapy based on low or high-grade mitotic and Ki-67 indexes, and there is limited benefit for prognostication, with some utility in evaluating for recurrence. There is a lack of consensus on the diagnostic and prognostic role of Ki-67 index in pulmonary carcinoids [30,31,32].

There is currently not an adequate consensus on the role of surgery in metastatic disease [3,4,5]. The impact of metastatic disease, with or without resection, does not change survival benefits. In our review, one study demonstrated a statistically significant worse prognosis with the use of adjuvant therapy in the metastatic nodal setting with resection [12]. Other studies evaluating the role of adjuvant therapy in metastatic nodal disease, regardless of resection status, did not dictate a difference in outcomes [19,20,21]. This is not to disfavor the use of palliative chemotherapy for the relief of symptoms involved with tumor burden when appropriate.

#### 4.1.3. Current Guideline Based Therapy

Current guidelines do not call for adjuvant therapy in typical carcinoids, consistent with our review overall and one study showing a disadvantage. NCCN guidelines support the use of chemotherapy in atypical carcinoids [8]. These recommendations are partially based on literature showing the response of pulmonary carcinoids treated with any chemotherapy [19,33,34]. The studies cited in guideline recommendations for use of chemotherapy in pulmonary carcinoids are generalized from chemotherapy response in small cell lung cancer (SCLC), per the NCCN [8,35,36,37]. Due to the seldom-seen nature of pulmonary carcinoid, and more specifically atypical carcinoid, performing a prospective trial to find the optimal treatment is difficult and lacking. While histologically and pathologically similar to other neuroendocrine cancers, there is a lack of evidence to demonstrate that regimens extrapolated across cancer types are applicable to improving survival benefits.

Different societies have recommendations for somatostatin analogs. The NCCN and ENETS recommend somatostatin analogs (SSA) use in patients with well-differentiated NETs in certain circumstances if positive on radionucleotide scan [5,8]. ENETS also recommends use in patients with a Ki-67 ≤ 10% along with a positive somatostatin receptor status [5]. ESMO along with NANETS also supports the use of octreotide labeled analogs in symptomatic patients with positive somatostatin receptors [9,10]. Of note, slower more well-differentiated tumors correlated to a longer OS and PFS [3].

### 4.2. Genomics of Carcinoid Tumors

#### 4.2.1. Profiling of Carcinoid Tumors

Genomic and and immunohistochemical analysis comparing pulmonary NET’s is lacking [38]. Thought to be different histological subtypes by low and high grade (TC and AC vs. LCNEC and SCLC), new literature examining the status of these tumors indicate that rather than distinct etiologies, advanced secondary high-grade NET’s may arise from preexisting carcinoids on a spectrum. This is evident in genomic and transcriptomic data revealing ACs as tumors representing both TCs and LCNECs and SCLCs. Recent multi-omic studies have attempted to molecularly delineate these tumors further than histological types [34,35].

TC and AC display driver mutations in approximately 73% of cases, mainly MEN1 and SWI/SNF complex subunit mutations (ARID1A, SMARC1, SMARCA2, SMRCA5). Less common are RB1 and TP53 mutations [38,39] This correlates with data from other NGS studies of NETs with ACs [40,41,42]. Another analysis classifying 31 TC and 11 AC showed that 40% of sequenced tumors elucidated mutations in histone modifier genes such as: MEN1, ARID1A AND PSIP1 with 20% with SWI/SNF complex mutations, correlating with previously mentioned studies [43]. A large Chinese study with 18 TCs and 24 ACs used a larger 520 gene panel showing LCNEC (12.7 mutations/mb) harbored more mutations than SCLC (11.9 mutations/mb), ACs (7.1 mutations/mb), and TC’s (2.4 mutations/mb). SCLC and ACs displayed 26.3% and 20.8% of driver mutations seen in LCNEC demonstrating increasing evidence for a wide scope of disease and mutations burden between subtypes with similar molecular profiles.

#### 4.2.2. Genomic Spectrum of Carcinoids

Rather than historical classification based off histological subtypes, there is now evidence that a molecular spectrum may help to further subclassify NETs. Work from Simbolo et al. using molecular analysis classified subtypes of pulmary NETs into different clusters which they correlated to the grade C3—low (AC) to C1—high (LCNEC)). The lower C3 cluster profiling mainly histological Acs, demonstrated low MEN1 and high RB1 expression, consistent with the literature. Other studies have shown that while historically TC and AC were considered distinct from higher grade NETs such as LCNEC and SCLC, molecularly ACs tend to behave more LCNEC like in terms of progression of disease [44]. This must be contrasted with analysis showing distinct differences between carcinoids (TC, AC) and SCLC, with little difference between TC and AC [43]. There are still lacking and conflicting data as much of these analyses were limited in their breadth of analysis. There likely are other genomic alterations that would benefit from whole exome sequencing to elicit more targeted molecular therapies.

## 5. Conclusions

Surgical intervention has been demonstrated and continues to be the primary treatment of pulmonary carcinoids. Based on the available data presented in the literature, there is no current evidence to support the current guidelines of adjuvant chemotherapy after surgical resection of pulmonary carcinoid tumors for typical or atypical carcinoids. In particular cases, SSA use may be appropriate, while in more aggressive and advanced tumors support care can be utilized. Nodal status and tumor staging also do not play a significant role in survival. Due to the uncommon nature of this malignancy, it is difficult to conduct a formal prospective trial. With whole genome sequencing, a logical next step is to identify molecular mutations for a more targeted therapy that could possibly be used in the adjuvant setting. While clinical judgment can be exercised concerning when chemotherapy may be favorable, specifically in palliative care, there is no current literature to support the use of adjuvant chemotherapy versus observation after resection.

## Figures and Tables

**Table 1:** Articles selected assessing survival benefit of individuals with typical and/or atypical carcinoid who underwent surgical resection and received adjuvant chemotherapy. Abbreviations: Progression-Free Survival (PFS); Overall Survival (OS); Median Overall Survival (MOS); Typical Carcinoid (TC); Atypical Carcinoid (AC); S—Surgery; S + CT—Surgery + Chemotherapy.

Article1	Type of Study	Study Analysis	TC and AC (n)	Nodal Status(n)	Survival Analysis	*p* Value	Study Conclusions
Wegener [14] The role of adjuvant therapy for atypical bronchopulmonary carcinoids (2019)	Retrospective Review	Analysis of NCDB: patients with stage I-III atypical carcinoid treated surgically +/- adjuvant therapy	TC—N/A AC—67	I/II-38 III-29	Median survival: With chemotherapy 63 months Without Chemotherapy 79 months	0.89	No benefit with adjuvant therapy
Nussbaum [11] Defining the role of adjuvant chemotherapy after lobectomy for typical bronchopulmonary carcinoid tumors (2014)	Retrospective Review	Analysis of NCDB for patients who underwent lobectomy for typical carcinoid with metastatic disease treated with adjuvant chemotherapy	TC—37 AC—N/A	I/II/III-37	Survival at 5 years: Chemotherapy—69.7% No chemotherapy—81.9%	0.042	Use of adjuvant chemotherapy is associated with worse overall survival among both unadjusted and propensity matched groups with typical carcinoid that are nodal positive Propensity score matching showed inferior survival that was not statistically significant
Anderson [12] Adjuvant Chemotherapy Does Not Confer Superior Survival in Patients with Atypical Carcinoid Tumors (2017)	Retrospective Review	Analysis of NCDB to determine whether a survival advantage exists in patients receiving chemotherapy for pN0 or pN+ atypical carcinoid tumors	TC—N/A AC–104	pN0—15pN+—89	Adjuvant Chemotherapy: 12 mo—98.9% 60 mo—47.9% Surgery alone: 12 mo—98.4%, 60 mo—67.1%	0.46	Use of adjuvant chemotherapy postoperatively in patients with pN+ and pN0 disease conferred no survival advantage
Song [13] Long-term outcomes and prognostic factors of patients with surgically treated pulmonary atypical carcinoid tumors: our institutional experience with 68 patients (2018)	Retrospective Review	All patients with a diagnosis of primary pulmonary AC tumor treated from 1999 to 2013 were reviewed.	TC—N/A AC—31	N/A	RFS and OS between those who received adjuvant chemotherapy after resection and those who had operation alone (*p* = 0.957 and *p* = 0.718, respectively	0.957 and 0.718	The use of adjuvant chemotherapy postoperatively in patients with pathologically lymph node-positive and pathologically lymph node-negative disease seems to have no survival advantage.
Gosain [15] Role of Adjuvant Chemotherapy in Pulmonary Carcinoids: An NCDB Analysis (2019)	Retrospective Review	Using codes for TC and AC in the National Cancer Database (NCDB), all surgically resected cases from 2004–2014 were included to evaluate the need for adjuvant chemotherapy	6673 cases were included: TC—88% AC—12%	N/A	TC patients did well with surgery alone in all Stages AC patients: Stage 1–5-year OS with S vs. S + CT: 84% and 52% Stage II—5 year OS with S vs. S + CT: 81% and 55% Stage III—5-year OS with S vs. S + CT: 46% vs. 54%	N/A	No benefit was seen from adjuvant chemotherapy in TCs. Adjuvant therapy may add benefit in stage III AC, the results were not statistically significant
Huang [16] Assessment of the prognostic factors in patients with pulmonary carcinoid tumor: a population study (2018)	Retrospective Review	Cases of pulmonary carcinoid tumors were extracted from the Surveillance Epidemiology and End Results database.	N/A	N/A	N/A	N/A	Multivariate analyses showed that radiotherapy and chemotherapy were negative prognostic factors
Tancredi [17] The Post-Surgical Long-Term Behaviour of Lung Carcinoid Tumours (2015)	Retrospective Review	Retrospective evaluation of long-term behaviour of lung carcinoids after surgery. A total of 23 patients (17 with typical pulmonary carcinoids and 6 with atypical pulmonary carcinoids) were enrolled in our hospital from April 1994 to July 2009	TC-2	N/A	N/A	N/A	There is no role for adjuvant and neoadjuvant chemotherapy in typical and atypical carcinoid
Furqan [18] Lobar versus sub-lobar surgery for pulmonary typical carcinoid, a population-based Analysis (2018)	Retrospective Review	The Surveillance, Epidemiology, and End Results (SEER) Program was used to select patients ≥66 years old, and diagnosed between 2000 and 2012 with pulmonary TC.	N/A	N/A	N/A	N/A	Role of adjuvant CTX and XRT is unclear as these did not improve survival in this study The number of patients who received adjuvant CTX was small, we did not see survival advantage from it
Chong [19] Chemotherapy and irradiation for locally advanced and metastatic pulmonary carcinoid tumors (2014)	Retrospective Review	Analysis of typical and atypical carcinoid tumors treated between 1990 and 2012	7—not specified	N/A	N/A	N/A	The small number of patients receiving adjuvant treatment and the long duration of follow-up needed in this disease makes it difficult to draw conclusions on the impact this approach has in the survival of patients with resected disease
Forde [20] Systemic Therapy, Clinical Outcomes, and Overall Survival in Locally Advanced or Metastatic Pulmonary Carcinoid: A Brief Report (2014)	Retrospective Review	The Johns Hopkins Pathology Database was reviewed for APC patients treated between January 1992 and December 2012. Data on time to recurrence, progression-free survival, and overall survival were estimated by using the Kaplan–Meier method.	N/A	N/A	Response rate and PFS for each therapy were estimated using both radiology and clinical notes and thus reflect real-life practice; however, as a consequence have a degree of associated uncertainty.	N/A	Both TC and AC tumors demonstrate response to platinum/etoposide chemotherapy and we consider this to be a standard first-line option at our institution
Robelin [21] Characterization, Prognosis, and Treatment of Patients with Metastatic Lung Carcinoid Tumors (2019)	Retrospective Review	Retrospectively analyzed the medical records MLC patients treated at two tertiary referral centers in France (Gustave Roussy Institute—EURACAN center, and Hospices Civil de Lyon—ENETS center of excellence) from November 1995 to June 2017.	108—type not specified	N/A	PFS Platin/Etoposide n = 24: 7.1 months. Oxaliplatin n = 84: 9.3 months. MOS Platin/Etoposide n = 24: 44 months. Oxaliplatin n = 84: 37.8 months.	N/A	Further studies are required to define the therapeutic strategy that would most benefit patients

**Table 2:** Recommendations and grade of evidence on adjuvant therapy for pulmonary carcinoid (TC and AC) from: National Comprehensive Cancer Network (NCCN; North American Neuroendocrine Tumor Society (NANETS); European Society of Medical Oncology (ESMO); European Neuroendocrine Tumor Society (ENETS) [5,8,9,10].

	NCCN	NANETS	ESMO	ENETS
ADJUVANT THERAPY	Atypical: Observation or Cytotoxic Chemotherapy	No Recommendation	No Recommendation	Consider in AC with high proliferative index
GRADE OF EVIDENCE	2B	N/A	N/A	Level IV

**Table 3:** Summary of Ki-67 indices in pulmonary and non-pulmonary carcinoids from selected studies.

Boland [23]				
	Median Ki-67	Ki-67 Range	*p*-value (N/A)	
Typical (n = 41)	1.6	0–10.7		
Atypical (n = 14)	4.3	1.2–12.2		
Marchió [24]				
	Ki-67 < 4% (n = 147)	Ki-67 < 4–9% (n = 60)	Ki-67 ≥ 10% (n = 32)	*p*-value (0.02)
Homogenous	80	32	18	
Heterogeneous	13	8	11	
Childs [29]				
	Ki-67 ≤ 2%	Ki-67 3 ≤ 20%	Ki-67 > 20%	*p*-value (0.002)
	18	80	75	N = 173

## Data Availability

These data were presented at the 2021 NCCN Conference. https://jnccn.org/view/journals/jnccn/19/3.5/article-pBPI21-009.xml (1 September 2022).
